# Supplementary material for: Comprehensive Evolutionary Analysis of the SMXL Gene Family in Rosaceae: Further Insights into Its Origin, Expansion, Diversification, and Role in Regulating Pear Branching
Source: Int J Mol Sci. 2024 Mar 4;25(5):2971. doi: 10.3390/ijms25052971 (PMC10931622; doi:10.3390/ijms25052971)
Supplement: Supplementary file 1 [file ijms-25-02971-s001.zip › FIigure S1.pdf]

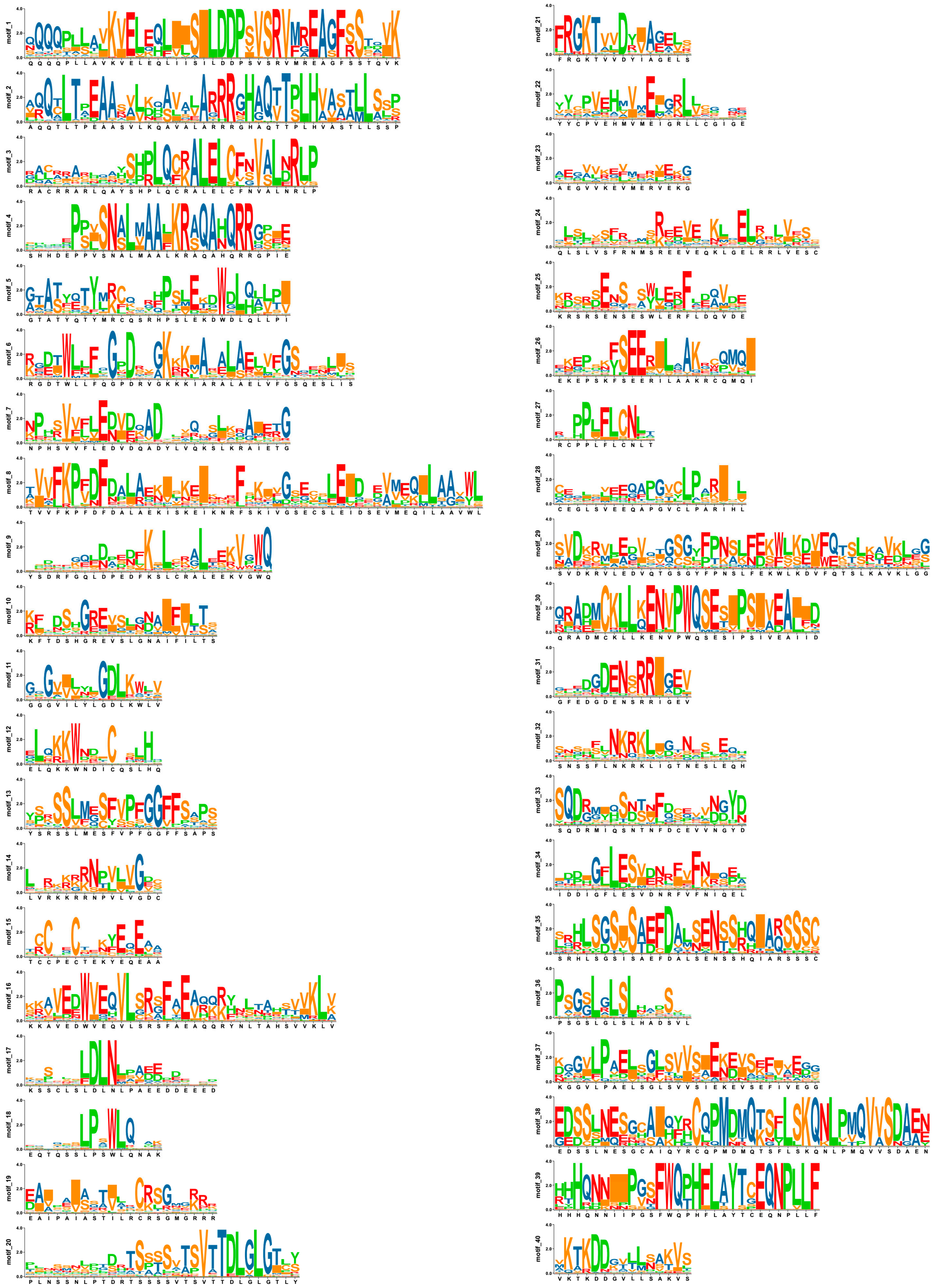

Figure S1. PpySMXL motif structure amino acid position. Residual conservation for all proteins is shown by the height of each letter.
